# Student Perceptions of Fee Policies in Veterinary Medicine: An Ethical and Professional Identity Framework

**DOI:** 10.3390/vetsci13090845

**Published:** 2026-08-22

**Authors:** Aysun Gültekin, Pınar Ayvazoğlu-Demir

**Affiliations:** 1 Department of Veterinary History and Deontolog, Faculty of Veterinary Medicine, Adnan Menderes University, 09016 Aydın, Türkiye; aysunkoc@hotmail.com; 2 Department of Livestock Economics and Management, Faculty of Veterinary Medicine, Kırıkkale University, 71450 Kırıkkale, Türkiye

**Keywords:** economic sustainability, deontology, price competition, regulatory compliance, professional socialization

## Abstract

This study was conducted to examine the perceptions and attitudes of final-year veterinary students regarding the Official Minimum Fee Schedule established for veterinarians in Türkiye, within a multidimensional framework. The research was conducted with a total of 216 final-year students enrolled in veterinary faculties in Türkiye. Data were collected using a questionnaire consisting of Likert-type items. The construct validity of the scale was assessed using Exploratory Factor Analysis (EFA), and its reliability was measured using Cronbach’s alpha coefficients. The study found that the majority of students considered providing services below the official minimum fee schedule to be ethically inappropriate and reported that professional organizations play an important regulatory and supervisory role in this regard. In terms of demographic variables, no statistically significant differences were found in factor scores based on gender, income level, university, level of education, or internship experience. However, significant differences were identified in the dimensions of regulatory–deontological attitudes and price transparency based on place of residence. In conclusion, attitudes toward the minimum fee schedule in veterinary medicine are shaped not only by legal knowledge but also by ethical values, professional socialization processes, and institutional norms. These findings highlight the importance of addressing fee policies in veterinary education together with their ethical and professional dimensions, as well as strengthening the awareness-raising role of professional organizations.

## Simple Summary

The prices charged for veterinary services can affect both the sustainability of veterinary practices and access to animal healthcare. In Türkiye, veterinarians are expected to follow an official minimum fee schedule, making pricing not only an economic issue but also a matter of professional responsibility. Understanding how future veterinarians view these rules may help identify how professional values develop during veterinary education. Our findings show that final-year students generally recognize the ethical importance of complying with the fee schedule and support professional oversight and price transparency. These findings suggest that economic aspects of veterinary practice should be addressed alongside ethics and professional responsibilities during undergraduate education.

## 1. Introduction

The veterinary profession plays a critical role in animal health, public health, and environmental sustainability. However, the economic sustainability of veterinary practices has emerged as a significant challenge, particularly for veterinarians at the beginning of their careers [1]. In Türkiye and elsewhere, fee structures are among the key determinants of long-term professional stability and workforce sustainability [2]. Although ethical and professional regulations aim to safeguard professional integrity and prevent unfair competition [3,4], practice conditions often deviate from these standards. Early-career veterinarians frequently face low starting salaries, irregular income, and heavy workloads [5]; when combined with rising student debt and economic uncertainty, this situation can undermine motivation, service quality, and long-term professional commitment [6]. Kipperman et al. [1] reported that small animal veterinarians frequently encounter ethical dilemmas due to financial constraints. Groves et al. [7] demonstrated that veterinarians attempt to manage this tension by offering treatment options at different cost levels instead of a single “gold standard”. Quain et al. [8] noted that financial limitations during times of crisis exacerbate ethically challenging situations and contribute to moral stress. These findings indicate that the conflict between service fees and owners’ payment capacity, as well as the tension between professional responsibility and economic pressures, not only affects ethical decision-making and professional satisfaction but also directly shapes the formation of professional identity during students’ socialization process [9,10].

Economic regulations are theoretically discussed as being intertwined with ethical values and shaping professional identity; fee schedules, on the one hand, aim to prevent unfair competition and protect professional prestige, while on the other hand, they raise ethical questions such as justice, accessibility, and the payment capacity of animal owners [11]. It has been reported that veterinarians frequently encounter ethical dilemmas due to financial constraints, which directly affect their decision-making processes [12]. Similarly, Williams [13] reported that early-career veterinarians may experience difficulties in balancing treatment options with the financial limitations of animal owners, which can affect their professional wellbeing, clinical practice, and learning experiences.

In Türkiye, the official minimum fee schedule established by the Turkish Veterinary Medical Association (TVHB) serves as a regulatory tool aimed at maintaining economic and professional standards in veterinary service provision [14]. Non-compliance with the official minimum fee schedule may have deontological and disciplinary implications under the relevant professional regulations [14,15]. However, compliance with these rules remains limited in practice, raising concerns regarding professional ethics and service quality [15]. More broadly, professional accountability in veterinary medicine extends beyond clinical practice and encompasses adherence to established ethical, legal, and professional responsibilities [16]. Studies conducted in Türkiye indicate that unfavorable income and economic conditions among veterinarians are associated with lower job satisfaction and concerns regarding professional sustainability [17,18,19]. International studies indicate that students’ career choices are influenced by both income expectations and non-material factors such as work–life balance [20,21]. In Türkiye, however, income level has been identified as an important determinant of professional satisfaction [22].

It is emphasized that the formation of professional identity during the socialization process of veterinary students is shaped by the internalization of professional values [9]. In this context, the role of economic regulations is of critical importance; students and young veterinarians develop their professional identity based on how they perceive these regulations and how they internalize professional values. Kimera and Mlangwa [10] demonstrated that students’ acquisition of ethical sensitivity and economic awareness directly influences their professional decision-making processes in the future.

This study assumes that students’ perceptions of the Official Minimum Fee Schedule will be decisive in shaping their professional identity formation and ethical sensitivity. Accordingly, the research examines the perceptions of final-year veterinary students in Türkiye regarding the fee schedule and aims to evaluate the issue as a scientific problem within the context of ethical dilemmas and professional identity formation. In this way, it seeks to provide a scientific contribution to understanding how economic regulations intersect with professional values and ethical responsibilities.

## 2. Materials and Methods

This study was conducted among final-year students at the Faculties of Veterinary Medicine of Aydın Adnan Menderes University and Kırıkkale University. The questionnaire was administered online to all eligible students, and participation was voluntary. The required sample size was calculated using Cochran’s formula with a 5% margin of error and 95% confidence level. After applying finite population correction, the minimum sample size was determined as 218 participants [23]. Following data screening, two incomplete questionnaires were excluded, and analyses were performed on 216 valid responses.

The questionnaire consisted of two sections: (i) socio-demographic variables (gender, residence, income level) and (ii) items measuring perceptions and expectations regarding the official minimum fee schedule. Attitudes were assessed using a five-point Likert scale (1 = strongly disagree, 5 = strongly agree). A pilot study with 20 students (≈10% of the target sample) was conducted to assess clarity and content validity. Minor revisions were made before final data collection.

IBM SPSS Statistics 21.0 was used for data analysis. he assumption of normal distribution was assessed using the Shapiro–Wilk test prior to conducting parametric tests. Differences between groups were examined using the independent-samples *t*-test and one-way analysis of variance (ANOVA). Statistical significance was set at *p* < 0.05. Construct validity was assessed using the Kaiser–Meyer–Olkin (KMO) measure and Bartlett’s sphericity test, followed by Exploratory Factor Analysis with Principal Component extraction and Varimax rotation. Internal consistency was evaluated using Cronbach’s alpha, and α ≥ 0.70 was considered acceptable [24].

## 3. Results

### 3.1. Socio-Demographic Characteristics of the Participants

A total of 216 final-year students participated in the study. The sample was balanced by university (Kırıkkale: 48.1%; Aydın Adnan Menderes: 51.9%) and gender (female: 51.4%; male: 48.6%). The majority (85.6%) reported a monthly family income below USD 2500. Most participants (62.5%) were raised in provincial centers, whereas 37.5% grew up in districts or rural areas.

In the study, a large majority of students (88.4%) were found to have internship or observational experience in private veterinary clinics. Although awareness of the official minimum fee schedule was high (82.9%), a substantial proportion of students (67.1%) evaluated the current tariff as inadequate.

### 3.2. Factor Structure and Reliability Analysis

Descriptive statistics for the 11 Likert-scale items are presented in Table 1. Mean scores ranged from 3.88 to 4.67, with standard deviations between 0.74 and 1.24, indicating generally high levels of agreement regarding the importance of tariff compliance and professional regulation. The highest agreement was observed for the statement that providing services below the minimum fee constitutes a deontological violation (4.67 ± 0.74), whereas the lowest mean score was related to strict personal compliance if opening a clinic (3.88 ± 1.01).

The suitability of the data for factor analysis was confirmed by a KMO value of 0.788 and a significant Bartlett’s test of sphericity (χ^2^ = 567,104; df = 55; *p* < 0.001). Reliability analysis yielded a Cronbach’s alpha of 0.765 (0.773 for standardized items), indicating acceptable internal consistency.

Exploratory Factor Analysis (Principal Component Analysis with Varimax rotation) initially identified a four-factor structure with eigenvalues greater than 1. One item with low and cross-factor loadings (<0.40) was removed, and the analysis was repeated. The final four-factor solution explained 64.82% of the total variance, with the first factor accounting for 32.51%. Eigenvalues and explained variance are presented in Table 2.

As shown in Table 2, a four-factor structure with eigenvalues greater than 1 was obtained. The first factor accounted for 32.51% of the total variance, while the four factors together explained 64.82% of the total variance. Following Varimax rotation, the factor analysis revealed that the items clustered under four distinct factors. The rotated factor loadings are presented in Table 3.

As shown in Table 3, primary factor loadings ranged from 0.518 to 0.832. Examination of the factor structure indicated that Factor 1 comprised items related to ethical compliance, sanction mechanisms, and professional supervision; Factor 2 included perceptions regarding price competition, collegial solidarity, and service quality; Factor 3 encompassed items associated with compliance with the official fee schedule and professional prestige; and Factor 4 reflected expectations related to price transparency and regulatory updates. Item 10 was excluded from the analysis due to its low factor loading (<0.40) and inverse correlation pattern. Item 1 loaded primarily on Factor 1 (0.654), while also showing a secondary loading on Factor 4 (0.446); therefore, it was retained under Factor 1 based on its higher primary loading.

The Regulatory and Deontological Attitudes subscale demonstrated acceptable reliability (α = 0.747), while the Competition and Professional Impact Perception (α = 0.660) and Price Transparency and Regulatory Expectations (α = 0.605) subscales showed moderate reliability. In contrast, the Fee Schedule Compliance and Professional Prestige subscale, consisting of two items, exhibited low internal consistency (α = 0.355).

### 3.3. Comparison of Factor Scores According to Demographic and Experiential Variables

No statistically significant differences were observed in factor scores according to gender across any subscale (*p* > 0.05). Effect size estimates indicated negligible to small practical differences. Similarly, factor scores did not differ significantly by monthly family income level (*p* > 0.05).

When analyzed by place of residence, statistically significant differences were identified for Regulatory and Deontological Attitudes (Factor 1) and Price Transparency and Regulatory Expectations (Factor 4) (*p* < 0.05). However, post hoc comparisons did not reveal significant pairwise differences between residence groups. No significant differences were found for Competition and Professional Impact Perception (Factor 2) or Fee Schedule Compliance and Professional Prestige (Factor 3).

Comparisons based on university, awareness of the minimum fee schedule, and internship experience in private veterinary clinics showed no statistically significant differences across any factor (*p* > 0.05). Effect sizes ranged from negligible to small, indicating limited practical impact of these variables on students’ attitudes.

## 4. Discussion

The organizational structure of veterinary services influences clinics’ decision-making processes and professional autonomy [9]. Professional ethics constitutes the normative framework governing professional conduct and plays a central role in shaping decision-making processes in veterinary practice [10]. In Türkiye, veterinary service fees are regulated through official minimum fee schedules [14]. In contrast, professional organizations such as the AVMA and RCVS provide ethical and professional guidance rather than establishing mandatory minimum or maximum veterinary fees [25]. These differences indicate that the relationship between fee-setting and professional ethics should be interpreted within the specific regulatory and professional context of each country.

In institutional veterinary practice, performance-based targets may intensify conflicts between service quality and pricing decisions, thereby raising the likelihood of ethical dilemmas in clinical settings [10]. Our study revealed that students do not perceive the official minimum fee schedule merely as an economic instrument; rather, they evaluate it within a framework related to ethical norms, competitive pressures, and professional prestige. This demonstrates that fee policies are not limited to economic regulations but are intertwined with broader professional value systems. Consequently, attitudes toward pricing policies are shaped not only by economic factors but also by ethical and professional values, consistent with research showing that veterinary students develop awareness of ethical and professional responsibilities during their education [25]. Moreover, Quain et al. [26] reported that financial constraints during times of crisis exacerbate ethically challenging situations and generate moral stress, which indicates that students’ evaluation of fee policies within an ethical framework is directly related to professional identity formation.

In this study, the high proportion of students who considered providing services below the official minimum fee schedule ethically inappropriate was found to be consistent with the literature suggesting that such situations may be associated with economic pressures and competitive conditions [27,28,29,30]. However, some studies have reported that ethical dilemmas in pricing are often linked to clients’ financial constraints and payment capacity [12,31,32,33]. Unlike human health systems, where costs are generally insurance-based, in veterinary medicine financial responsibility largely falls on pet owners; this makes pricing decisions ethically more sensitive [32]. Furthermore, considering the literature emphasizing that fee policies highlight the ethical dimension of professional sustainability and findings showing that students internalize ethical values during their education, it can be argued that students’ perceptions are not only shaped by economic pressures but also at the intersection of ethics, economics, and practice in the formation of professional identity. Veterinarians, like physicians, benefit from advanced diagnostic and therapeutic technologies; however, high-cost interventions require balancing animal welfare with clients’ payment capacity, which can complicate the ethical decision-making process [34,35]. Our findings indicate that deviations from standard fee schedules in the financial sustainability of veterinary clinics raise ethical concerns [36,37]. The fact that the majority of students (82.9%) are aware of the official minimum fee schedule, while a significant proportion (67.1%) consider it inadequate, suggests that students may have concerns regarding the adequacy of current fee levels in relation to their future professional practice. This interpretation is supported by Williams [13], who reported that early-career veterinarians may experience difficulties in balancing appropriate veterinary care with clients’ financial limitations, with potential consequences for their wellbeing, clinical practice, and learning experiences. These findings suggest that financial issues encountered in veterinary practice may have implications extending beyond economic concerns to the professional experiences of veterinarians. Therefore, providing veterinary services below the official minimum fee schedule may increase economic pressure on veterinarians and negatively affect their professional motivation [26,38,39].

In the study, factor loadings above 0.40 indicate that students perceive violations of fee policies as elements that could damage the professional image of veterinary medicine. This finding is consistent with studies suggesting that stricter fee regulation systems (such as those reported in the United Kingdom) are associated with more clearly defined ethical boundaries [33]. However, Gautier [40] offers a different perspective, noting that ethical violations are often explained in financial terms and may become normalized over time. Therefore, the present findings suggest that students’ perceptions are shaped by the level of regulation and professional context, while also highlighting the need to consider opposing views that emphasize the legitimization of ethical violations through economic reasoning.

In this study, an acceptable level of internal consistency (α = 0.747) was obtained for the “Regulatory and Deontological Attitudes” subscale. This result suggests that students’ ethical approaches to wage policies exhibit a consistent structure. The fact that students expressed the need for more emphasis on ethical issues in the curriculum supports the importance of ethics education in strengthening professional attitudes. Indeed, the literature also indicates that ethical judgment does not develop spontaneously through experience alone, but that structured education is necessary in this process [31,41].

In Europe, professional veterinary ethics is regarded as one of the fundamental components of undergraduate veterinary education [41]. The level of ethical and deontological awareness identified in this study indicates the importance of ethics education in students’ professional decision-making processes. However, the limited tools available to measure ethical competence require that the findings be interpreted with caution [42,43,44]. The results suggest that ethics education is not merely the transmission of knowledge but also a process that supports the adoption of professional values; this is consistent with studies in the literature emphasizing the necessity of structured ethics education [31,41].

The analysis in this study revealed no statistically significant gender differences across the subscales, indicating that attitudes toward fee policies and ethical regulations are not directly influenced by gender. This finding is consistent with studies reporting no gender-based differences in professional decision-making or conflict management [33,41]. Nevertheless, although some literature refers to gender-related differences in moral stress or ethical perception [1,45], and in certain contexts female students scoring higher in moral reasoning [42], the present data do not support these views. Similarly, the absence of significant differences in factor scores according to students’ income levels suggests that ethical and regulatory approaches develop independently of economic background; this finding is also consistent with previous student-based studies [46]. In contrast, significant differences in factor scores according to students’ place of residence suggest that socio-environmental conditions may influence ethical and regulatory perceptions. Higher scores among students raised in provincial centers may reflect greater exposure to institutional and professional settings. Indeed, previous research has emphasized that demographic and socio-cultural contexts play an important role in shaping professional orientations and value perceptions [18,47].

The “Competition and Perceived Professional Impact” factor was found to exhibit moderate internal consistency (α = 0.660). This indicates that students share similar views regarding the effects of price competition on professional quality and peer relationships. Accordingly, students believe that price competition may negatively impact professional standards in the long term. This finding is consistent with other studies showing that competitive pressure can weaken peer relationships and reduce service quality [10,33]. More broadly, economic and market pressures represent important challenges for contemporary veterinary practice [48]. Furthermore, veterinarian density has been shown to influence veterinarians’ income, suggesting that competition within the veterinary services market may also have important economic implications [49]. The low Cronbach’s alpha observed for the two-item Fee Schedule Compliance and Professional Prestige subscale (α = 0.355) should be interpreted cautiously, as reliability estimates may be affected by the limited number of items in very short scales [50].

The analysis results revealed no statistically significant differences based on knowledge level or internship experience. This finding indicates that ethical attitudes are shaped not only through formal coursework but also through students’ observations and experiences during the educational process. This process aligns with the concept of the “hidden curriculum,” which refers to the values students unconsciously acquire in the educational environment [51,52]. Previous studies have also reported changes in value orientations during veterinary education [52], supporting the view that ethical perceptions may develop through both formal education and experiential and environmental influences [44].

The analysis revealed no statistically significant differences among universities across the subscales (*p* > 0.05). This indicates that students’ perceptions of ethical and regulatory norms are shaped within a common professional framework. The role of regulatory structures, professional organizations, and similar educational experiences in the formation of professional identity is emphasized in the literature [9,53]. However, the continuity of these norms is not limited to undergraduate education; post-graduation experiences and market conditions also play a significant role [20,38]. In this context, strengthening ethical and economic norms requires not only curricular efforts but also continuous institutional and professional support. This approach is consistent with studies highlighting the multidimensional nature of ethical development in professional education [54] and with international regulations emphasizing the continuity of ethical responsibilities in veterinary training [55].

## 5. Conclusions

This study showed that final-year veterinary students in Türkiye perceive the Official Minimum Fee Schedule not solely as an economic instrument but also in relation to ethical values, professional identity, and institutional norms. Most students considered providing services below the schedule ethically unacceptable and emphasized the importance of professional supervision and regulatory mechanisms. These findings highlight the importance of addressing the ethical and economic dimensions of fee policies within veterinary education and strengthening the awareness-raising role of professional organizations to support professional responsibility and the long-term sustainability of veterinary practice.

## Figures and Tables

**Table 1:** Descriptive statistics of the items included in the Likert scale.

Items	Mean	SS
Providing services below the minimum fee is a deontological violation.	4.67	0.74
The minimum fee schedule protects professional prestige.	4.37	1.08
Competition among private veterinarians hinders tariff compliance.	4.21	0.88
Price competition weakens collegial solidarity.	4.26	0.86
Price competition reduces long-term professional quality.	4.27	0.93
I would comply strictly with the minimum fee schedule if I opened a clinic.	3.88	1.01
Violations of the fee schedule should be subject to clear and deterrent sanctions.	4.27	1.03
Private veterinarians should be more strictly supervised by TVHB regarding tariff compliance.	4.49	0.80
This issue should receive greater emphasis in veterinary education.	3.91	1.24
Service prices should be publicly displayed in veterinary clinics.	4.35	0.89
Deontological regulations should be revised in line with free market principles.	4.14	0.96

**Table 2:** Total Variance Explained Eigenvalues > 1.

Factor	Eigenvalue	Explained Variance (%)	Cumulative (%)
1	3545	32,513	32,513
2	1377	12,515	45,028
3	1170	10,635	55,663
4	1008	9160	64,823

Extraction Method: Principal Component Analysis.

**Table 3:** Rotated Component Matrix for the 12-item Scale (Varimax Rotation).

No	Scale Item	Factor 1	Factor 2	Factor 3	Factor 4
1	Providing services below the minimum fee constitutes a deontological violation.	0.654	–	–	0.446
2	Sanctions applied in cases of fee schedule violations should be clear and deterrent.	0.800	–	–	–
3	Self-employed veterinarians should be subject to stricter supervision by the TVHB.	0.726	–	–	–
4	Greater emphasis should be placed on this topic in the educational curriculum.	0.719	–	–	–
5	Competition among self-employed veterinarians makes compliance with the minimum fee schedule more difficult.	–	0.518	–	–
6	Price competition undermines collegial solidarity.	–	0.711	–	–
7	Price competition reduces professional quality in the long term.	–	0.832	–	–
8	The minimum fee schedule protects professional prestige.	–	–	0.799	–
9	If I were to open a clinic, I would comply with the minimum fee schedule.	–	–	0.632	–
11	Prices should be made visible in veterinary clinics.	–	–	–	0.752
12	Deontological provisions should be updated.	–	–	–	0.700

## Data Availability

The raw data supporting the conclusions of this article will be made available by the authors on request.
